# Autism spectrum quotient, coping with stress and quality of life in a non-clinical sample – an exploratory report

**DOI:** 10.1186/s12955-015-0370-x

**Published:** 2015-10-26

**Authors:** Ewa Pisula, Dorota Danielewicz, Rafał Kawa, Wojciech Pisula

**Affiliations:** Faculty of Psychology, University of Warsaw, Warsaw, Poland; Institute of Applied Psychology, The Maria Grzegorzewska College for Special Education, Warsaw, Poland; Institute of Psychology, Polish Academy of Science, Warsaw, Poland

**Keywords:** Autistic traits, AQ, Quality of life, Coping styles, Emotion-oriented coping, Social diversion

## Abstract

**Background:**

It has been shown that autistic traits may be observed both in individuals with autism spectrum disorders and to a lesser extent in the general population. Since these traits are closely associated with limitations in social functioning, they make development of interpersonal relations difficult, and therefore may have a negative impact on an individual’s quality of life (QoL). The purpose of this study was to explore these links, including the mediating effects of coping styles.

**Methods:**

A sample of 154 adults aged 19–38 years completed three questionnaires: Autism Spectrum Quotient (AQ), Coping Inventory for Stressful Situations and World Health Organization Quality of Life—BREF. Pearson’s r correlation coefficients were analysed, followed by path analysis.

**Results:**

All domains of QoL (Physical health, Psychological, Relationships and Environmental) were negatively correlated with AQ. The correlations were low or moderate (from −0.36 to −. 42). AQ was also correlated with two coping styles: positively with Emotion-oriented coping and negatively with Social diversion. Path analysis models showed the mediating effect of coping styles with respect to the relationships between autistic traits and QoL domains. Autistic traits and coping styles explained the greatest level of variance for the Psychological domain (41 %).

**Conclusions:**

The results confirmed the relationship between autistic traits and QoL, mediated by two coping styles. Due to the small sample and narrow age range of participants, our findings should be treated as a preliminary report.

## Background

Autism spectrum disorders (ASD) are life-long developmental disorders characterized by social and communication impairments and restricted patterns of behaviour and interests [1, 2]. Due to their specific characteristics, especially their highly negative impact on the ability to establish social relations, ASD often lead to poor social adjustment and poor long-term outcomes with respect to independent living [3, 4]. This is true even in high-functioning individuals with ASD, i.e. those without intellectual disabilities [5, 6].

Autistic characteristics affect the functioning of the individual in a way that may adversely influence subjective quality of life (QoL). The World Health Organization (WHO) defines subjective QoL as an individual’s perception of their position in life in the context of the culture and value systems in which they live and in relation to their goals, expectations, standards and concerns [7]. Subjective QoL is a multifaceted concept including, among other things, subjective perception of physical health and psychological state, social relationships and salient features of the environment. Thus defined, subjective QoL has been demonstrated to be lower in individuals with ASD than in the general population [8]. It has been also found that individuals with Asperger syndrome (one of the disorders grouped within ASD) were more likely to report depression, suicidal ideation, suicide plans and attempts compared not only to the general population, but also those with medical or psychic illnesses [9].

Many researchers share the view that certain behavioural and cognitive characteristics typically seen in autism, referred to as autistic traits or Broader Autism Phenotype [10, 11], are present not only in individuals with ASD and their first-degree relatives, but are also distributed throughout the general population [12–15]. The dimensional nature of ASD is still up for debate, and it is unclear whether autistic traits in individuals with ASD and those without such a diagnosis are the same phenomenon [16, 17]. Nevertheless, extensive research is being conducted in non-clinical groups to explore links between autistic traits and other aspects of human psychological functioning. These studies often utilize the Autism Spectrum Quotient questionnaire (AQ) developed by S. Baron-Cohen et al. [14].

Links between autistic traits and subjective QoL have not been fully elaborated yet, despite the arguments existing for undertaking such research. Autistic traits, by definition, may inhibit the development of satisfying social relationships. Specifically, it has been demonstrated that individuals with greater expression of autistic traits reported stronger feelings of loneliness [18, 19], depression and anxiety, as well as higher rates of being bullied [20]. In a study of newlywed couples, Pollmann, Finkenauer and Begeer [21] found that higher levels of autistic traits in males were associated with lower relationship satisfaction. Considering the importance of interpersonal relations for individual wellbeing [22], the relationships between autistic traits and QoL certainly deserve attention from researchers.

It has also been established that the relationships between personal characteristics and QoL are mediated by coping with stress [23–25]. Lazarus and Folkman [26] defined coping with stress as cognitive and behavioural efforts undertaken to meet specific external and internal demands that individuals perceive as stretching or exceeding their resources. Depending on an individual’s personality characteristics, certain coping styles may affect subjective QoL differently [23]. To our knowledge, there is currently no information about links between autistic traits and coping with stress in non-clinical samples. These traits may increase the risk of experiencing stress and impair the use of effective coping methods, since difficulties in social relations resulting from autistic traits may hinder the use of coping styles that involve seeking support from others. Furthermore, neuroticism, which is positively correlated with autistic traits [27, 28], is known to be strongly associated both with perceived stress [29] and coping with stress [30]. To date, however, the relationships between autistic traits, coping styles and QoL have not been specifically targeted in research. The aim of the present study is to identify and describe these relationships in a non-clinical sample of young adults. The concepts of autistic traits, as well as a Broader Autism Phenotype (BAP) or AQ, may imply slightly distinct theoretical notions, and as stand-alone constructs are deserving of detailed empirical analysis. However, due to preliminary nature of this study, we have decided to limit our analysis involving the measurement of autistic characteristics to the Autism Spectrum Quotient concept, reflected by the total score from the AQ questionnaire [14]. We expect to find negative correlations between AQ and QoL, and to confirm the mediating role of stress-coping styles in the relationships between these variables. Since our limited knowledge makes it impossible to specify the nature of these relationships more precisely, especially with respect to coping styles, this is an exploratory study in its nature.

## Methods

### Sample

The participants group consisted of 153 individuals (93 females, 60 males) aged 19–38 years (M = 22.11 years, SD = 2.10 years). They were university students of humanities, social sciences, economics and science.

### Instruments

#### Autism Spectrum Quotient (AQ)

AQ is a self-report survey to assess autistic traits in a general population. It consists of 50 statements to which participants respond on a 4-point Likert scale (1- definitely agree, 2- slightly agree, 3- slightly disagree, 4 - definitely disagree). One point is awarded for each diagnostic answer, which is “agree” in one half of the statements and “disagree” in the other half (irrespective of whether “slightly” or “definitely”), therefore reducing the four point scale to dichotomous one, as proposed by Baron-Cohen et al. [14]. The total score ranges from 0 to 50 points, with higher scores representing greater severity of autistic traits. It should be noted that using the conventional 0–1 scoring procedure and reducing the scale from a four-point to a two-point range may be questionable. We decided to do so out of a desire to make our data compatible with the vast majority number of previous studies.

AQ allows for the calculation of the total score and five subscale scores: Social skill, Communication, Attention switching, Imagination and Attention to detail. Some studies have shown that while reliability for the total score is acceptable, it is significantly lower for some of the subscales [13, 28, 31–33]. Furthermore, the hypothesized five-factor structure of AQ has yet to be confirmed empirically [13, 34–36]. As described earlier [30] the Polish version of the Autism-Spectrum Quotient was translated from English into Polish with the consent of Prof. Simon Baron-Cohen by Ewa Pisula, Ph.D., Agnieszka Rynkiewicz, MAT, M.D. and Izabela Łucka, M.D., Ph.D. Next, a different translator back-translated the Polish version AQ into English. The original English and back- translated versions were compared by a native English speaker. The text was edited according to comments received from a native English speaker, following which the reformulated items were translated into English by a translator unfamiliar with the content of the comments and original questionnaire then compared with the original by a native English speaker; the final Polish version of the AQ was then established (available at http:/www.psychologia.pl/rehabilitacja/, as well as from the first author upon request). The Polish version of AQ employs the same scoring system as the original. The psychometric properties of this instrument were analysed [32]. Reliability (AQ total score) measured using Cronbach’s alpha coefficient in a Polish sample (2819 individuals) has been found satisfactory, *r* = 0.71 [32], although—as in studies conducted in other countries cited above—the internal consistency coefficients for subscales (excepting social skill) were low. This is why only the total AQ score is analysed in the present study. It should be mentioned that the absence of the subscales in our analyses creates obvious limitations in interpretation of the results. However, given the exploratory nature of our study, sample size and unsatisfactory psychometric properties of the subscales [32], we chose not to conduct a detailed analysis involving AQ subscales.

#### Coping Inventory for Stressful Situations (CISS)

CISS measures coping styles [37]. It consists of 48 items comprising three scales: (1) Task-oriented coping—engaging in efforts aimed at solving a problem through cognitive restructuring or attempts to alter the situation and solve the problem; (2) Emotion-oriented coping—responding to stress with self-oriented emotional reactions, focusing activity on the reduction of emotional tension caused by the stressor; (3) Avoidance-oriented coping—tendency to avoid a stressful situation in one of two ways: avoidance by social diversion and avoidance by distraction.

Items are answered on a 1 (not at all) to 5 (very much) Likert scale. The Polish version of CISS was used, which features high reliability ranging from 0.78 to 0.9 for individual subscales [38].

#### World Health Organization Quality of Life—BREF (WHOQOL-BREF)

The subjective QoL measure was the WHOQOL-BREF, which is derived from the 100-item WHOQOL [7]. The instrument comprises 26 items, including two general questions regarding (1) subjective overall perception of QoL and (2) individual, overall perception of the subject’s health, as well as 24 belonging to four domains: physical health (7 items), psychological state (6 items), social relationships (3 items) and environment (8 items). Participants respond to each item on a 5-point scale (1 = very poor/very dissatisfied/not at all; 2 = poor/dissatisfied/a little; 3 = neither poor nor good/a moderate amount; 4 = good/satisfied/very much; 5 = very good/very satisfied/extremely). The study used the Polish version of WHOQOL-BREF prepared by Jaracz [39]. Scores in each of the four domains were analysed (the general questions were ignored). Scores for individual domains were calculated by computing the arithmetic mean for the items belonging to a given domain and multiplying the result by four (to obtain scores comparable with the full version of WHOQOL). The Polish version of WHOQOL-BREF measured by Cronbach’s alpha is satisfactory. The values for individual domains are 0.81 for Physical, 0.78 for Psychological, 0.69 for Relationships and 0.77 for Environmental [39].

#### Demographics survey

In the demographics survey the participants were asked to state their age, gender and field of study. They were also asked if they had ever been diagnosed with ASD. In addition, they were asked whether they had close relatives diagnosed with childhood autism, Asperger syndrome or pervasive developmental disorders not otherwise specified.

### Data collection and ethics

The study was approved by the Ethics Committee of the Faculty of Psychology at the University of Warsaw. The study was administered in groups; all participants were informed about the purpose of the study, voluntary participation and anonymity. The order of scales in the sets distributed to students was randomized. Incomplete questionnaires (7 out of 160) were rejected before analysis. The number of participants given above (*N* = 153) includes only the sets qualified for analysis. None of the participants declared that they had ever been diagnosed with ASD. Three participants declared that members of their extended family (cousins) had been diagnosed with Asperger syndrome.

### Statistical analysis

Statistical calculations were performed using SPSS/AMOS v. 21. Assessment of links between AQ, coping styles and QoL domains employed Pearson’s product moment correlation coefficients, followed by path analysis. The mediating effects of coping styles on the relationship between AQ and QoL were tested using path analysis models. The maximum likelihood estimation method was used. The adjusted level of significance, in general α/k for k tests, was used to conduct each of the individual k tests and was applied to tested models when there was at least two predictors of the same variable.

## Results

### Descriptive findings

The descriptive statistics of the scores obtained by the students in all scales used in the study are shown in Table 1.

**Table 1:** Descriptive statistics of AQ, QoL and coping styles. Means and Standard Deviations are shown both across gender groups and the total sample

	Females		Males		Total sample						
Variable	Mean	Standard deviation	Mean	Standard deviation	Mean	Standard deviation	Min	Max	Range	Skewness	Kurtosis (centered at 3)
AQ	15.76	5.59	17.32	6.98	16.37	6.20	1.00	36.00	35.00	0.45	3.28
QoL domains											
Physical	16.31	2.07	16.80	2.03	16.50	2.06	9.14	20.00	10.86	−0.66	3.99
Psychological	13.64	2.56	14.33	2.60	13.91	2.59	5.33	18.67	13.34	−0.58	3.33
Relationships	15.47	3.51	15.47	3.64	15.47	3.55	5.33	20.00	14.67	−0.69	2.75
Environmental	14.29	2.22	14.90	1.88	14.53	2.11	7.00	19.00	12.00	−0.37	3.32
Coping styles											
Task-oriented coping	59.03	7.78	58.62	7.27	58.87	7.56	30.00	77.00	47.00	−0.59	4.59
Emotion-oriented coping	47.13	11.65	43.38	8.81	45.66	10.75	17.00	74.00	57.00	0.18	3.16
Avoidance-oriented coping	49.17	8.78	45.07	8.49	47.56	8.87	29.00	76.00	47.00	0.53	3.30
Avoidance by distraction	21.81	5.71	20.03	5.26	21.11	5.59	9.00	36.00	27.00	0.44	3.01
Avoidance by Social diversion	18.37	3.84	16.50	3.56	17.63	3.83	6.00	25.00	19.00	−0.24	3.23

Mean differences between gender groups in “Emotion-oriented coping” and “Avoidance by Social diversion” are statistically significant. The t-test values are 2.258 and 2.882 respectively, resulting respectively in *p* < 0.05 and *p* < 0.001

### Correlation analysis

As the initial step for further analysis, Pearson’s product moment correlation coefficients were calculated. The results are shown in Table 2.

**Table 2:** Correlations of the AQ with the quality of life domains and coping styles

Correlations	AQ total
1. Physical domain	- 0.37^*^
2. Psychological domain	- 0.42^*^
3. Relationships domain	- 0.36^*^
4. Environmental domain	- 0.41^*^
5. Task-oriented coping	- 0.07
6. Emotion-oriented coping	0.36^*^
7. Avoidance-oriented coping	- 0.18
8. Social diversion	- 0.42^*^
9. Distraction	0.04

^*^ - *p* < 0.001 (Correction of *p* was made according to the following formula: 0.05/9 comparisons = 0.006)

All measured aspects of QoL were negatively correlated with total AQ score. The correlations were low or moderate (from −0.36 to −0.42). In addition, the AQ score correlated with two coping styles: positively with Emotion-oriented coping and negatively with Social diversion.

### Path analyses

**Fig. 1:**
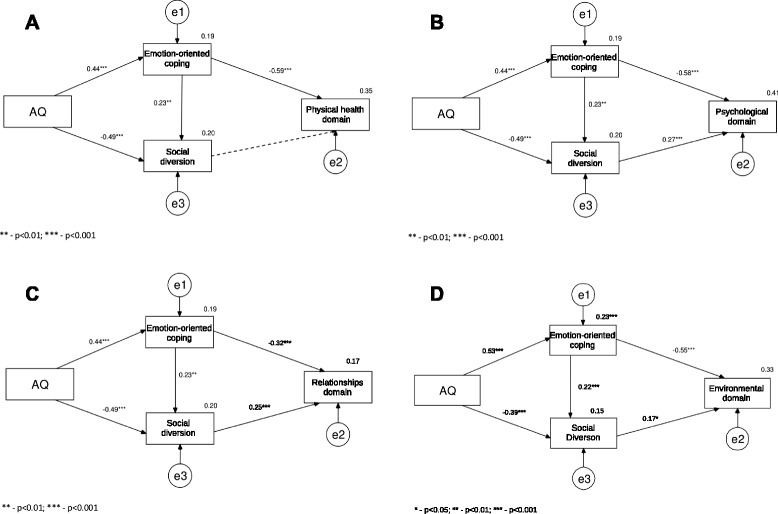
Path analysis models for QoL domains: (**a**) – Physical health domain; (**b**) – Psychological domain; (**c**) – Relationships domain, and (**d**) – Environmental domain

Possible mediators included in the path analysis models were Emotion-oriented coping and Social diversion, i.e. those coping styles for which statistically significant relationships with AQ were found. Each QoL domain was analysed in a separate model. For the sake of nonnormality of the Relationships domain distribution (Kolmogorov-Smirnow Z = 2.07, *p* < 0.001), this variable was log-transformed. The transformation was successful and facilitated proceeding with further analysis. The results of the path analysis models for each QoL domain are presented in Fig. 1.

As Fig. 1 shows, Emotion-oriented coping was a full mediator of the relationship between AQ and each of the QoL domains. AQ predicted Emotion-oriented coping, which in turn was negatively correlated with QoL.

In the model for the Physical health domain of QoL (see Fig. 1a), AQ explained 19 % of variance in Emotion-oriented coping, while Emotion-oriented coping in turn explained 35 % of Physical health domain variance. Bonferroni correction was applied to test the significance of the relationships between Emotion-oriented coping, Social diversion and QoL domains. There was no statistically significant association between Social diversion and Physical health, meaning that Social diversion was not a mediator of the relationship between AQ and the Physical health domain. One path with no significant relationship was dropped and it is presented in Fig. 1a for illustrative purposes only. Based on modification indices values and in order to achieve a better fit of the model, the path representing Social diversion as an effect of Emotion-oriented coping was added. Controlling for AQ revealed the association between Emotion-oriented coping and Social diversion. AQ was negatively related to Social diversion, while Emotion-oriented coping was positively correlated with Social diversion. Both predictors together explained 20 % of the variance in Social diversion.

There was no statistically significant difference between the tested model and data, χ^2^ (2) = 2.03, *p* > 0.05. The fit indexes had appropriate values, CFI = 0.99, RMSEA = 0.01. The Sobel test has shown that the effect of Emotion-oriented coping as a mediator of the relationship between AQ and Physical health domain was statistically significant, Z = − 4.67, *p* < 0.001. Emotion-oriented coping mediated 70.2 % of the total effect of AQ on the Physical health domain.

In the model presented in Fig. 1b, Social diversion was positively correlated with the Psychological domain of QoL. Emotion-oriented coping and Social diversion were two mediators of the relationship between AQ and QoL. AQ was positively correlated with Emotion-oriented coping and negatively related to Social diversion. The Psychological domain was negatively correlated with Emotion-oriented coping and positively related to Social diversion. Based on the modification indices values, in order to achieve a better fit of the model the path representing Social diversion as an effect of Emotion-oriented coping was added, the same as in the model for the Physical health domain. Emotion-oriented coping was positively related to Social diversion, which, when taken together with AQ, explained 20 % of Social diversion variance. The two mediators of Emotion-oriented coping and Social diversion explained 41 % of Psychological domain variance. There was no statistically significant difference between the tested model and the data, χ^2^(1) = 2.89, *p* > 0.05. The fit indexes had appropriate values for this model as well, CFI = 0.99, RMSEA = 0.11. The Sobel test has indicated that the effect of Emotion-oriented coping as a mediator of relationship between AQ and Psychological domain was statistically significant, Z = − 4.49, *p* < 0.001. The value of the Sobel test for Social diversion tested as mediator was not statistically significant, Z = − 1.30, *p* > 0.05. Emotion-oriented coping mediated 46.6 % of the total effect of AQ on the Psychological domain.

The results for the Relationships domain are presented in Fig. 1c. The model was very similar to that obtained for the Psychological domain, but both Emotion-oriented coping and Social diversion explained only 17 % of the Relationships domain. There was no statistically significant difference between the tested model and the data, χ^2^ (1) = 2.65, *p* > 0.05, and the fit indexes had appropriate values, CFI = 0.98, RMSEA = 0.01. The Sobel test has shown that the effect of Emotion-oriented coping as a mediator of relationship between AQ and the Relationships domain was statistically significant, Z = − 2.31, *p* < 0.01. The value of the Sobel test for Social diversion tested as mediator was not statistically significant, Z = −1.59, *p* > 0.05. Emotion-oriented coping mediated 26.7 % of the total effect of AQ on the Relationships domain.

The model for the Environmental domain is shown in Fig. 1d. This model was very similar to those obtained for the Psychological and Relationships domains. The relationship between Social diversion and the Environmental domain was weaker than for the Psychological and Relationships domains, however, both Emotion-oriented coping and Social diversion explained 33 % of the Environmental domain. There was no statistically significant difference between the tested model and the data, χ^2^ (1) = 3.48, *p* > 0.05, while the fit indexes had values of CFI = 0.98, RMSEA = 0.13. The increased RMSEA value is an effect of the weaker correlation between Social diversion and the Environmental domain. The Sobel test has shown that the effect of Emotion-oriented coping as a mediator of the relationship between AQ and Environmental domain was statistically significant, Z = − 4.24, *p* < 0.001. The value of Sobel test for Social diversion tested as mediator was not statistically significant, Z = − 0.09, *p* > 0,05. Emotion-oriented coping mediated 48.92 % of the total effect of AQ on the Environmental domain.

Equivalence of the causal structures between the groups of men and women was also tested. The χ^2^ differences were not statistically significant: for Physical health Δχ^2^ (4) = 0.01, *p* > 0.05, for the Psychological domain Δχ^2^ (5) = 0.01, *p* > 0.05, for Relationships Δχ^2^ (5) = 0.01, *p* > 0.05 and for the Environmental domain Δχ^2^ (5) = 0.01, *p* > 0.05, so it can be assumed that the regression paths operate equivalently across the two groups in each model.

## Discussion

As expected, our study identified relationships between AQ and QoL. AQ was correlated with all QoL domains. While these correlations were low to moderate, any interpretations should take into account the small sample size. The anticipated direction of the relationship was confirmed: higher levels of AQ correlated with lower QoL. This is consistent with the results of studies on subjective QoL in individuals with ASD and their parents [8, 40]. Mugno et al. [40] showed that the parents of children with ASD tended to report lower QoL than the parents of children with cerebral palsy and intellectual disabilities. The authors of the paper cited above claim that their results may be interpreted not only in terms of specific difficulties experienced by parents raising children with ASD, but also as an effect of the genetic predispositions to develop both autistic traits and depression, and the impact of these factors on the reported QoL. It is possible that the relationship between autistic traits and susceptibility to depression, which other authors have also hinted at [41–43], has affected the results of the present study as well. This interpretation is supported by some empirical data [44] and opens up the interesting prospect of exploring neurobiological mechanisms common to autistic traits and depression, although this issue lies outside the scope of this paper. Since this controversy cannot be resolved on the basis of the data presented in the present study, we must leave this question open for future research. The negative correlation between AQ and QoL can result from the impairment of social relations caused by autistic traits. Autistic traits are associated with lower satisfaction with social relationships and with a sense of loneliness [18, 21]. There is evidence that individuals with higher levels of autistic traits are at a higher risk of rejection and aggression from others [27], which in turn can affect their subjective evaluation of QoL.

Correlation analysis revealed the presence of associations between AQ and two coping styles: Emotion-oriented coping and Social diversion. Autistic traits seem to be positively associated with a tendency to use coping styles focused on emotions and negatively with a tendency to cope by using Social diversion. In the case of the correlation between AQ and Emotion-oriented coping, we can hypothesize that autistic traits may lead to the use of less adaptive ways of coping, thereby making access to some effective stress-coping styles more difficult (e.g. enlisting the help of others). It has been established that emotional-oriented coping is less adaptive and associated with poor QoL [45]. It is also noteworthy that research on individuals with ASD and members of their families, i.e. along with the dimensional approach to ASD, has demonstrated that intense stress triggers stereotyped behaviour, rituals, self-stimulation and aggressive behaviour, as well as crying and anger, which is thought to reduce emotional arousal [46]. The positive correlation between AQ and emotion-focused coping may indicate that individuals with higher levels of AQ use emotion concentrated coping to reduce emotional arousal. It may be noted that some studies have shown a relationship between autism spectrum traits and alexithymia [47, 48]. Alexithymia, defined as difficulty in identifying and describing one’s emotional experience [49], is associated with poor ability to regulate emotional arousal efficiently, and therefore low adaptive coping [50]. Positive correlations between alexithymia and repressive coping style, denial and disengagement have been already identified (eg. [51, 52]). Given the above, the positive correlation between AQ and emotion-oriented coping seems to be consistent with the outcomes of previously cited studies.

The link between AQ and Social diversion seems rather straightforward. This subtype of Avoidant coping implies engaging in social contact in order to avoid stressors. In people who tend to be significantly less involved in social relationships, this type of coping with stress is by definition less likely. Less frequent Social diversion coping was also observed in parents of children with ASD [53]. It remains unclear whether this is due to time constraints that prevent these parents from spending time in the company of others, or whether it is somehow related to the level of autistic traits in parents. We could expect people with higher levels of autistic traits to make less frequent use of coping styles that require involvement in social contacts, such as seeking social support. This issue, however, is beyond the scope of the present paper. It should be emphasized, moreover, that we found no correlations between AQ and other measured coping styles, including Task-oriented coping or the other subtype of the Avoidant style—Distraction.

An interesting aspect of the present study is the presence of the mediatory effect of Emotion-oriented coping and Social diversion on the relationship between autistic traits and QoL. The direction of the relationship with QoL is not surprising in the case of Emotion-oriented coping. Consistently with the findings of previous studies that demonstrated a negative correlation of this coping style with QoL [54, 55], it is negative for all QoL domains. The findings regarding Social diversion are less straightforward. The style turned out to be a predictor for all QoL domains except Physical health. In each of the remaining domains, the correlation between Social diversion and QoL was positive, i.e. the higher participants scored for that style, the higher QoL they reported. The developed models indicate that autistic traits lower the probability of using Social diversion as a coping style, and the style mediates the relationship between autistic traits and QoL. The avoidant style involving Social diversion proved to be beneficial in the context of QoL (although the correlation was weak). Avoidant coping is widely regarded as disadvantageous in terms of QoL [56–58]. Our findings suggest that it may be beneficial for the three measured areas of QoL. This could mean that for individuals with higher levels of autistic traits, coping by avoiding stressors through intensified socializing enhances their subjective QoL. It is noteworthy that a similar relationship between Avoidant coping and QoL has been found by other researchers, for example in patients after coronary angioplasty [59].

The percentage of variance explained by the analysed models differed across QoL domains. It was the highest (41 %) in the case of the Psychological domain. This domain includes, among others, acceptance of one’s bodily appearance, presence of negative feelings, overall satisfaction with oneself and self-esteem. The fact that the level of autistic traits reported by participants combined with coping styles explains a relatively high proportion of variance of QoL may reflect the significance of autistic traits in the context of various aspects of self-esteem. This issue, however, requires further research. The magnitude of the total effect of AQ on the QoL domains mediated by Emotion-oriented coping (from 26.7 % in Relationships to 70.2 % in Physical health) proved the validity of the coping styles input within the models of the AQ and QoL relations.

A relatively high proportion of QoL variance was explained by autistic traits and coping styles also with respect to the Physical health and Environmental domains (35 and 33 %, respectively). Physical health includes, among others, necessary medical treatment, assessment of one’s energy, ability to get around, pain and discomfort, assessment of sleep and ability to work. These are the key elements of the overall assessment of one’s health and physical condition. The scope of the Environmental domain is very broad: it includes, among other elements, financial resources, sense of freedom, physical and psychological safety, health and healthcare, living conditions, availability of new information and skills, opportunities for leisure and physical environment. The smallest proportion of QoL variance (17 %) was explained by AQ and coping styles in the case of the Relationships domain. This domain appears to be the one most directly linked with autistic traits, since it encompasses satisfaction with personal relationships, support from friends and sex life. Since in WHOQOL-BREF it consists of only three items, it provides very little information about subjects’ perceptions of their relationships with others. The limited scope of social relationships in the questionnaire may have affected the results in terms of variance in this QoL domain explained by autistic traits.

## Conclusions and limitations

Our findings have revealed links between autistic traits, coping styles and QoL, but they have also shown that variability of autistic traits in a general population sample is not a decisive factor when it comes to QoL. It is obvious that the latter is affected to a significant extent by other variables not measured in the present study. Any interpretation of the findings must take into account the limitations of the study, such as the size and characteristics of the sample: all participants were students of universities in large cities aged from 19 to 38 years. The study used exclusively self-report measures, which means that the participants themselves declared how many autistic traits they had, which coping styles they used and what their QoL was like. Any interpretation must also be limited to a non-clinical population only, since the relationship between autistic traits in the general population and in people with ASD has not yet been established, and our study did not involve participants with ASD.

It should also be noted that this study did not involve measurement of exposure to stress. This should be taken into account in future research, since autistic traits may affect perceived stress levels. Moreover, the possible role of neuroticism in shaping the links among AQ, coping with stress and QoL is worthy of analysis. Since neuroticism is associated with autistic traits [27, 28], as well as with perceived stress [29] and coping with stress [30], including all these variables into a tested model seems to be a promising idea.

Due to these limitations the present study should be treated as a preliminary report. Nevertheless, our findings can provide cues for further investigation of the factors that determine the relationships between autistic traits and perceived QoL. This may be especially important due to the rising incidence of ASD [60], which in turn may lead to new circumstances affecting the QoL of individuals who express those traits and their social partners.
